# 

**Published:** 1899-03-25

**Authors:** 


					raa nuornaju* ~~~ "The Standard of highest /purity."?Lanceu oubch.^&th, i8S3.
CADBURYS ^ CADBURYS 4
COCOA \ XTM Pv COCOA
ABSOLUTELY PURE. '==a NO DRUGS OR ALKALIES USED.
WWICH HOSP.LTAJb
Vol- XXV. Saturday,
Maboh 25th, 1899.
[SnbBonptionTerms.] pS,ICE TWOPENCE.

				

